# White matter abnormalities in the *Hdc* knockout mouse, a model of tic and OCD pathophysiology

**DOI:** 10.3389/fnmol.2022.1037481

**Published:** 2022-11-24

**Authors:** Kantiya Jindachomthong, Chengran Yang, Yuegao Huang, Daniel Coman, Maximiliano Rapanelli, Fahmeed Hyder, Joseph Dougherty, Luciana Frick, Christopher Pittenger

**Affiliations:** ^1^Department of Psychiatry, Yale University School of Medicine, New Haven, CT, United States; ^2^Department of Genetics, Washington University in St. Louis, St. Louis, MO, United States; ^3^Department of Radiology and Biomedical Imaging, Yale University School of Medicine, New Haven, CT, United States; ^4^Department of Biomedical Engineering, Yale University School of Medicine, New Haven, CT, United States; ^5^Yale Child Study Center, Yale University School of Medicine, New Haven, CT, United States; ^6^Interdepartmental Neuroscience Program, Yale University School of Medicine, New Haven, CT, United States; ^7^Center for Brain and Mind Health, Yale University School of Medicine, New Haven, CT, United States

**Keywords:** animal model, tourette syndrome, histamine, myelin, striatum

## Abstract

**Introduction:**

An inactivating mutation in the *histidine decarboxylase* gene (*Hdc*) has been identified as a rare but high-penetrance genetic cause of Tourette syndrome (TS). TS is a neurodevelopmental syndrome characterized by recurrent motor and vocal tics; it is accompanied by structural and functional abnormalities in the cortico-basal ganglia circuitry. *Hdc*, which is expressed both in the posterior hypothalamus and peripherally, encodes an enzyme required for the biosynthesis of histamine. *Hdc* knockout mice (*Hdc*-KO) functionally recapitulate this mutation and exhibit behavioral and neurochemical abnormalities that parallel those seen in patients with TS.

**Materials and methods:**

We performed exploratory RNA-seq to identify pathological alterations in several brain regions in *Hdc*-KO mice. Findings were corroborated with RNA and protein quantification, immunohistochemistry, and *ex vivo* brain imaging using MRI.

**Results:**

Exploratory RNA-Seq analysis revealed, unexpectedly, that genes associated with oligodendrocytes and with myelin production are upregulated in the dorsal striatum of these mice. This was confirmed by qPCR, immunostaining, and immunoblotting. These results suggest an abnormality in myelination in the striatum. To test this in an intact mouse brain, we performed whole-brain *ex vivo* diffusion tensor imaging (DTI), which revealed reduced fractional anisotropy (FA) in the dorsal striatum.

**Discussion:**

While the DTI literature in individuals with TS is sparse, these results are consistent with findings of disrupted descending cortical projections in patients with tics. The *Hdc*-KO model may represent a powerful system in which to examine the developmental mechanisms underlying this abnormality.

## Introduction

Tic disorders affect ∼5% of the population. Tourette syndrome (TS), which represents the severe end of the spectrum of tic disorders, affects 0.5–1% (Scharf et al., 2012; Scahill et al., 2013). Tic disorders are thought to derive from dysregulation of the cortico-striatal circuitry, and TS has been associated with anatomical and functional changes in this circuitry (Leckman et al., 2010; Williams et al., 2013). For example, early work documented slight but significant reductions in volume of the caudate and putamen (collectively known as the striatum) in both adults and children with TS (Peterson et al., 1993, 2003); reduced striatal volume in childhood predicts symptom persistence and severity in adulthood (Bloch et al., 2005). Abnormalities have also been reported in the thalamus (increased volume in TS) (Lee et al., 2006; Miller et al., 2010), corpus callosum (altered morphology) (Peterson et al., 1994; Plessen et al., 2004, 2006), and prefrontal cortex (mixed findings) (Peterson et al., 2001; Muller-Vahl et al., 2009). Diffusion tensor imaging (DTI), which uses water diffusion to measure white matter integrity, has revealed reduced fractional anisotropy (FA) in corticospinal pathways, internal capsule, inferior longitudinal fasciculus, and corpus callosum in youth with TS (Neuner et al., 2010; Yang et al., 2021).

The underlying causes of tics and TS have been slow to emerge and are likely to be complex and heterogeneous. One strategy to dissect pathogenesis in such complex conditions is to focus on rare disease-associated mutations of large effect size, which can be used as the basis for modeling pathophysiology in animals. A study of a two-generation pedigree with a high density of TS (as well as obsessive compulsive disorder, attention deficit disorder, and other commonly comorbid conditions) identified a nonsense mutation in the gene *histidine decarboxylase* (*Hdc*) as a candidate cause (Ercan-Sencicek et al., 2010). *Hdc* encodes the enzyme critical for biosynthesis of histamine (HA) from histidine, both in the brain and peripherally (Haas et al., 2008); this genetic finding thus suggests that HA deficiency may contribute to the pathophysiology of tics. Subsequent genetic studies have provided further evidence that HA dysregulation may contribute to TS, beyond this unique family (Fernandez et al., 2012; Karagiannidis et al., 2013).

We have studied *Hdc* knockout mice, which functionally recapitulate the original TS-associated mutation, as a potential model of tic pathophysiology. These mice exhibit repetitive behaviors, which may reflect some of the same circuit abnormalities as tics, after amphetamine challenge (Castellan Baldan et al., 2014) or stress (Xu et al., 2015), two manipulations that exacerbate tics in patients (Conelea and Woods, 2008; Denys et al., 2013; Buse et al., 2014). They also exhibit deficient sensorimotor gating, as do patients with TS (Castellan Baldan et al., 2014); this reinforces the conclusion that relevant brain networks are perturbed. *Hdc*-KO mice exhibit dysregulated dopamine tone and elevated neural activity in the striatum, as well as abnormalities in dopamine receptors that parallel those seen in TS patients carrying the *Hdc* mutation (Castellan Baldan et al., 2014). Together, these findings indicate that the *Hdc* KO mouse captures core components of the pathophysiology of TS and merits further study.

We conducted a small exploratory transcriptomic study of several brain regions in the *Hdc*-KO mouse model to generate hypotheses as to potentially pathogenic abnormalities. Unexpectedly, the most significant finding was an alteration in transcripts associated with oligodendrocytes and myelination in the basal ganglia. We used molecular, histological, and neuroimaging approaches to confirm the presence of white matter abnormalities in the dorsal striatum in this model of tic pathophysiology.

## Materials and methods

These experiments were carried out in accordance with ARRIVE guidelines.^1^

### Mice

*Histidine decarboxylase*-knockout mice have previously been described (Ohtsu et al., 2001); our mice are backcrossed to >N10 onto C57Bl/6 (Castellan Baldan et al., 2014). Mice were bred in-house at Yale University and Washington University in temperature and humidity-controlled vivaria, on a 12-h light/dark cycle. All experiments were approved by Institutional Animal Care and Use Committees (IACUC) at Yale University and Washington University and were consistent with the National Institutes of Health Guidelines on the Care and Use of Laboratory Animals. Euthanasia followed procedures approved by the Yale and Washington University IACUCs. For immunohistochemistry and neuroimaging, mice were deeply anesthetized and then transcardially perfused with PBS/paraformaldehyde. For RNA and protein preparation, mice were euthanized by cervical dislocation by appropriately trained personnel for rapid brain dissection. All other mice (e.g., breeders) were euthanized, when necessary, by CO_2_ asphyxiation. Adult male mice were used in all experiments.

### RNA-seq

2–3 month old *Hdc* knockout, heterozygous, and wild-type mice were euthanized and their brains rapidly dissected on ice. Ventral and dorsal striatum, hypothalamus, and motor cortex were rapidly dissected on ice, rapidly frozen, and stored at −80° until processing using RNeasy MinElute columns (Qiagen, Valencia, CA, United States). RNA quality and quantity were confirmed with a Bioanalyzer (Agilent Technologies, Santa Clara, CA, United States). We generated double-stranded complementary DNA using the Ovation RNA-Seq system V2 (NuGEN Technologies, Inc., San Carlos, CA, United States), starting from 5 ng of RNA. Standard sequencing libraries (Illumina, Inc., San Diego, CA, United States) were generated from 1 to 2 μg of cDNA, sheared to ∼200 bp. Sequencing was performed at the Genome Technology Access Center (GTAC) at Washington University in St. Louis.

Fastq files containing demultiplexed, single-end 50 base-pair reads were used as the basis for exploratory analysis. A total of 5′ and 3′ adapter sequences were trimmed from each read using Trimmomatic, and all reads shorter than 20 bases after adapter clipping were discarded (Bolger et al., 2014). rRNA reads were identified by mapping to a mouse rRNA reference sequence using Bowtie2 (Langmead and Salzberg, 2012) and were discarded. The remaining reads were aligned to the mouse genome (Ensembl GRCm38 annotation 75) using STAR (Dobin et al., 2013). SAMtools was used to convert SAM format to BAM format, then to create sorted and indexed BAM files for visualization (Li et al., 2009). Default union mode in HTSeq-count was used to compute read counts for reads uniquely mapping to exons in the mouse genome (Anders et al., 2015). In total, read depth was 266 M reads after counting, and reduced to 122 M total reads after QC; 3.5–5 M per sample. Genes expressed at low level (less than one copy per million reads) were filtered out. All RNA-Seq data in this study have been submitted to the Gene Expression Omnibus (GEO,^2^ accession #GSE161252).

### Pathway analysis

Differential gene expression was determined using the functions glmFit and glmLrt in the edgeR package from Bioconductor (Robinson et al., 2010). Gene ontology analysis was conducted using the gene ontology enrichment analysis and visualization tool (GOrilla),^3^ with one target and one background list of genes as the running mode (Eden et al., 2009). The target set was defined as all nominally upregulated genes in *Hdc*-KO mice for each tissue (*p* ≤ 0.05, uncorrected); the background set was defined as all genes expressed in *Hdc*-KO mice for each tissue. Cell-type specific expression analysis (CSEA) was conducted using a CSEA tool (Xu et al., 2014). The candidate list was defined as the genes up-regulated in *Hdc*-KO mice with false discovery rates (adjusted *p*-values) of less than 0.05.

### Quantitative polymerase chain reaction (qPCR)

Mice were euthanized and their brains rapidly dissected on ice; dissected tissue was rapidly frozen in liquid nitrogen. Tissue was homogenized in 1 mL TRIzol (Invitrogen, Waltham, MA, United States), and RNA was extracted following the manufacturer’s instructions. The PolyATtract mRNA Isolation System III (Promega, Madison, WI, United States) was used to isolate poly(A)^+^ mRNA. cDNA was synthesized using SuperSCript III First-Strand Synthesis SuperMix for qRT-PCR (Invitrogen). qPCR for oligodendrocyte-specific genes (see Supplementary Table 3) was performed in an Applied Biosystems 7500 Fast Real-Time PCR System using SYBR Green PCR Core Reagents (Applied Biosystems, Waltham, MA, United States), with final primer concentration of 0.3 μM each, and 4 μl cDNA in each 25 μl reaction. Specific cDNAs were quantified using standard curves, normalized to actin.

### Immunoblotting

Mice were rapidly euthanized, and tissue from striatum, cortex, and hippocampus was dissected on ice. Tissue was sonicated in radioimmunoprecipitation (RIPA) Lysis and Extraction Buffer (Thermo Fisher Scientific, Walthman, MA, United States) with cOmplete Protease Inhibitor Cocktail Tablets (F. Hoffman La Roche, Basel, Switzerland). Protein was quantified using a bicinchoninic acid (BCA) Protein Assay Kit (Thermo Fisher Scientific, Walthman, MA, United States). A total of 20 μg protein was added to diluted Laemmli Sample Buffer (Bio-Rad, Hercules, CA, United States) mixture (1x after mixing 9:1 with mercaptoethanol), denatured at 95° for 5 min, and loaded onto a 4–20% mini-PROTEAN TGX Precast Protein Gel (Bio-Rad) in Tris/glycine/SDS buffer, and separated at 100 V. Protein was transferred to polyvinylidene difluoride (PVDF) membranes at 35 V for 2 h in a buffer containing 10% vol/vol Tris/glycine buffer and 20% vol/vol methanol. Membranes were rinsed with tris-buffered saline (TBS) + 0.1% Tween-20, followed by Ponceau S solution (Sigma-Aldrich, St. Louis, MO, United States) and water, and then washed 3 × 10 min in TBS. Membranes were then blocked for 1 h in TBS + 0.1% Tween-20 with 5% non-fat dry milk, and then incubated at 4° overnight in the same solution with primary antibody: anti-MBP (1:1000, Millipore) or anti-MOBP (1:200, Santa Cruz Biotechnology, Santa Cruz, CA, United States). Membranes were then washed 2 × 10 min at room temperature in TBS + 0.1% Tween-20 and incubated for 1 h with HRP-labeled anti-mouse or anti-goat secondary antibody (1:10,000; Vector Laboratories, Newark, CA, United States). Chemiluminescence was visualized using the SuperSignal West Pico Chemiluminescent Substrate (Thermo Fisher Scientific, Walthman, MA, United States), visualized with the ChemiDoc XRS System (Bio-Rad). Quantification and analysis were performed using ImageJ (National Institutes of Health, Bethesda, MD, United States). Membranes were stripped for re-probing using Restore PLUS Western Blot Stripping Buffer (Thermo Fisher Scientific, Walthman, MA, United States) for 10 min. Loading was controlled with an anti-GADPH antibody (1:10,000; Millipore, Burlington, MA, United States).

### Immunohistochemistry

Mice were deeply anesthetized using ketamine/xylazine (100/10 mg/kg) and transcardially perfused with ice-cold PBS followed by ice-cold 4% paraformaldehyde (PFA) in PBS. Brains were dissected and post-fixed for 1 week in PFA at 4°C. Some of these brains were MR imaged (see below) before slicing.

Brains were equilibrated in 30% sucrose and sliced at 30 μm on a cryostat. Floating slices were stored at 4° in cryoprotectant (30% vol/vol glycerin and 30% vol/vol ethylene glycol). Slices were washed 3 × 10 min in PBS, endogenous peroxidases inhibited with 2% H_2_O_2_ for 20 min, washed 2 × 10 min in PBS, and then blocked for 30 min at room temp in PBS + 0.1% Triton X-100 + 2% normal goat serum. Slices were then incubated overnight at 4°C with the primary antibody [anti-APC (CC1) for oligodendrocytes, RRID:AB_443473, 1:1000, Abcam, Waltham, MA, United States; anti-MBP for myelin, RRID:AB_2140366, 1:1000, Millipore]. Slices were then washed 3 × 10 min in PBS + 0.1% Triton X-100, incubated with biotinylated goat anti-mouse secondary antibody (1:300, Vector Laboratories), washed 3 × 10 min in PBS + Triton X-100, and then developed using the VECTASTAIN Elite ABC HRP kit (Vector Laboratories), following the manufacturer’s instructions.

Slices were visualized on an upright Leica DM1000 microscope at 200x. Several images of the dorsal striatum were collected for each mouse, but only one field was collected for each section, to prevent overlap. For oligodendrocyte quantification, all stained oligodendrocytes in the captured image were counted. For myelin quantification, a threshold was set using ImageJ to identify area of myelin staining in each section. All immunohistochemical analysis was performed blind to condition.

### MR imaging

Perfused brains were post-fixed for 1 week in 4% PFA, as above. Brains were then washed for 3 × 10 min in PBS and then secured inside a custom MRI tube filled with heptacosafluorotributylamine (Fluorinert, Sigma-Aldrich, Inc., St. Louis, MO, USA). DTI was performed using a 9.4 T magnet (TR = 2,000 msec; TE = 25 msec; 1,000 msec RF pulses, 15 msec diffusion gradient duration, 5 msec delay) using a custom-made circular ^1^H surface radiofrequency transceiver coil (15 mm diameter), generating 26 coronal slices of 0.5 mm thickness. A total of 0.5 mm slices thickness is commonly used and provides optimal signal-to-noise ratio and tissue contrast within a reasonable imaging time (Chahboune et al., 2007, 2009a,b). Eighteen averages were acquired and the 128 × 64 images were zero-filled to 256 × 256, resulting in an in-plane resolution of 100 μm × 100 μm. Water diffusion was measured in 16 directions to compute the diffusion tensor (van Luijtelaar et al., 2013). FA was calculated and resulting parametric images were produced using BioImage Suite,^4^ with Gaussian smoothing (σ = 0.2 mm) of the individual FA images, followed by registration to an isotropic reference (100 μm) using non-linear warping (Duque et al., 2012). Given that the slices are contiguous (no gaps), resampling of DTI data to this finer resolution anatomical reference frame does not generate significant non-linear warping (Chua et al., 2009; Carlyle et al., 2012; Duque et al., 2012; Jung et al., 2016; Park et al., 2016; Johnson et al., 2018). Genotype groups were compared using whole-brain voxel-wise analysis.

### Statistics

Statistical analysis was performed using appropriate R packages for RNA-seq analysis, as detailed above, BioImage Suite for DTI data, and GraphPad Prism 7.0 for all other analyses. Between-group comparisons were performed by *t*-test or ANOVA; differences were considered significant at *p* < 0.05, after correction for multiple comparisons. All data are presented as mean ± SEM. For analysis of DTI data, unpaired two-tailed *t*-tests were used to determine voxel-wise statistical differences on FA maps.

## Results

### Transcriptomic analysis reveals upregulation of myelin-related genes in *histidine decarboxylase*-knockout mice

cDNA was prepared from four brain regions (dorsal and ventral striatum, hypothalamus, and motor cortex) of mice of three genotypes (wild-type [WT], *Hdc*-KO, and *Hdc* heterozygous) and sequenced on an Illumina platform. This was a pilot study intended for hypothesis generation; *N* was 2–3 per genotype and region, with a total of 266 M reads (3.5–5 M per sample, after mapping and filtering out low copies per million (CPM) transcripts; see Supplementary Tables 1–3). Multidimensional scaling of transcript profiles showed them to cluster by tissue, rather than by genotype (Supplementary Figure 1), as expected. The *Hdc* gene showed altered transcript levels, as expected.

**FIGURE 1:**
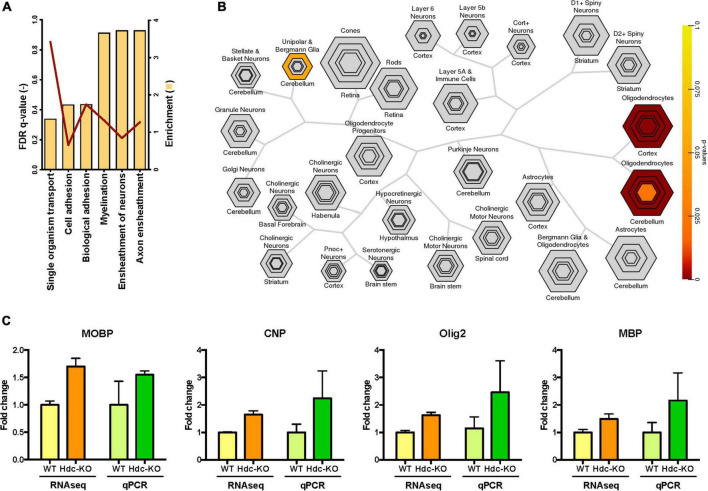
Striatally enriched genes in *histidine decarboxylase*-knockout (*Hdc*-KO) mice associated with white matter. **(A)** Gene ontology analysis (target set *n* = 778, background set *n* = 7376) revealed that the most enriched genes in the dorsal striatum (bars) were involved in axon ensheathment, ensheathment of neurons, and myelination. False discovery rate (FDR) corrected *q*-values are represented as lines. **(B)** Cell specific expression analysis (CSEA) comparing *Hdc*-KO with WT in the dorsal striatum (candidate gene list *n* = 40). Output is organized hierarchically by cell type to recapitulate biological relationships, with size corresponding to the size of the cell population and nested groups representing more specific populations to that cell type. Enriched genes were specific to oligodendrocytes. **(C)** Parallel changes in gene expression levels of four oligodendrocyte markers in the dorsal striatum in *Hdc*-KO mice by RNA-Seq and qPCR analysis.

We focused on a comparison of KO with WT transcriptomes; inclusion of heterozygotes gave qualitatively consistent but less clear results (not shown). The largest number of differentially expressed (DE) genes was seen in the dorsal striatum. We conducted Gene Ontology (GO) analysis of RNA-Seq results using GOrilla, to identify pathways that differ between genotypes. This analysis identified pathways in the DE genes that differed significantly between *Hdc*-KO and WT mice only in the dorsal striatum, not in the other tissues examined. Specifically, significant elevations were seen in the dorsal striatum in KO mice in genes associated with the biological processes of axon ensheathment, ensheathment of neurons, and myelination (Figure 1A and Supplementary Figures 2–4). This was driven by many genes known to be expressed in oligodendrocytes (Supplementary Figure 4), which led us to complement our GO analysis with an analysis focused on cellular composition.

Cell-type specific expression analysis is a strategy for inferring which cell types underlie alterations in tissue-specific gene expression (Xu et al., 2014). CSEA analysis in the dorsal striatum revealed upregulation of transcripts associated with oligodendrocytes in *Hdc*-KO mice (Figure 1B). We confirmed the findings from the RNA-seq analysis indicating increased expression of four oligodendrocyte markers (Myelin Basic Protein, MBP; Myelin Associated Oligodendrocyte Basic Protein, MOBP; Oligodendrocyte transcription factor 2, Olig2; and 2′,3′-Cyclic nucleotide 3′-phosphodiesterase, CNP; Supplementary Table 4) in the dorsal striatum by qPCR from the same samples; nominal increases in expression of all four genes were seen in *Hdc*-KO mice, supporting RNA-seq results (Figure 1C). Interestingly, no such increases were seen in an independent experiment using whole striatal homogenates, suggesting that the abnormality is restricted to dorsal striatum (Supplementary Figure 5). Overall, this screen suggested the presence of white matter abnormalities in the dorsal striatum of *Hdc*-KO mice.

### Increased expression of myelin-related proteins in *histidine decarboxylase*-knockout mice

**FIGURE 2:**
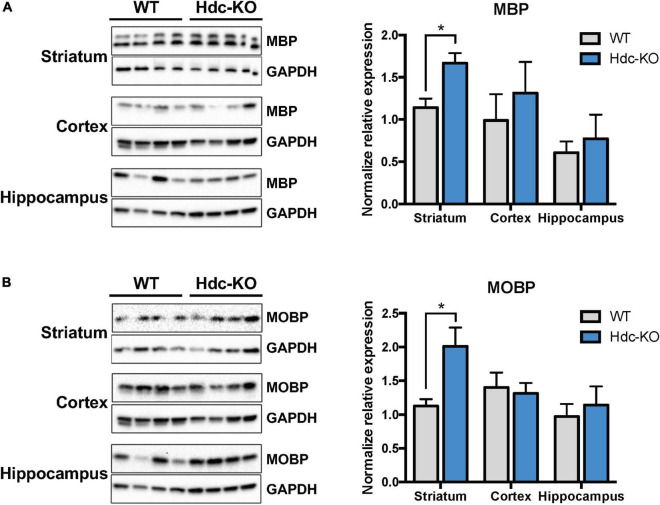
Increased myelin proteins in the striatum of *histidine decarboxylase*-knockout (*Hdc*-KO) mice. **(A)**. Western blot analysis showed elevated Myelin Basic Protein (MBP) in the striatum but not in the cortex or hippocampus of *Hdc*-KO mice, relative to WT controls. Striatum: *n* = 4, 4; *t*(6) = 3.33, *p* < 0.016; cortex, hippocampus n.s. **(B)** Similarly, Western blot analysis showed elevated Myelin Associated Oligodendrocyte Basic Protein (MOBP) in the striatum of *Hdc*-KO mice. Striatum: *n* = 4, 4; *t*(6) = 3.00, *p* < 0.025; cortex, hippocampus n.s. All data are presented as mean ± SEM. *p < 0.05.

RNAseq and qPCR assess transcriptional changes, but these do not necessarily translate into alterations in protein abundance. We therefore next tested the expression of myelin proteins-related in *Hdc*-KO and WT mice using immunoblotting. Both MBP (Figure 2A) and MOBP (Figure 2B) were upregulated in dorsal striatum of *Hdc*-KO mice, but not in cortex or hippocampus. This is consistent with the findings of RNA-seq and RT-PCR for these genes.

### Normal oligodendrocyte number but increased myelin cross-section in *histidine decarboxylase*-knockout mice

**FIGURE 3:**
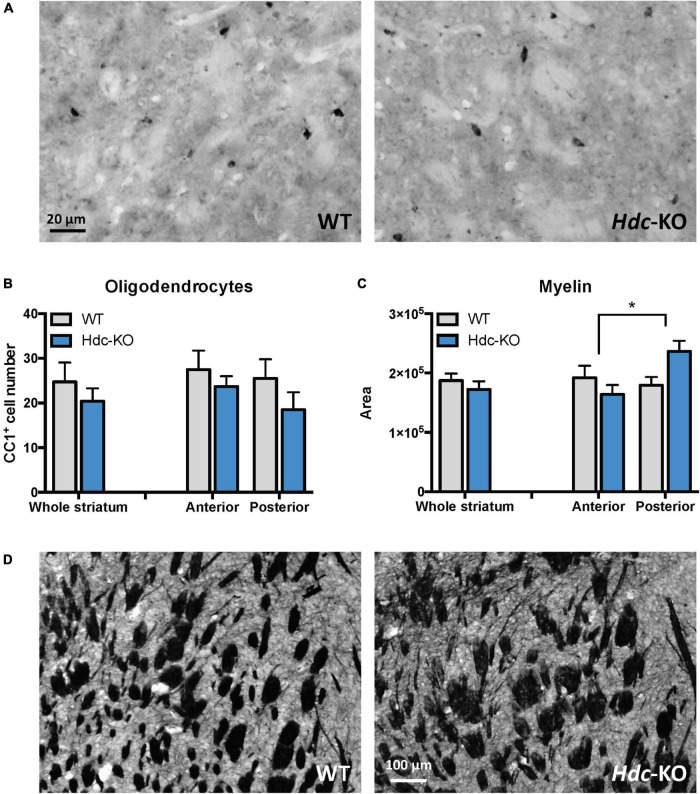
Oligodendroglia and myelin in *histidine decarboxylase*-knockout (*Hdc*-KO) mice. **(A)** Representative images of CC1 immunostaining of the striatum in wild-type and *Hdc*-KO mice. **(B)** There was no significant difference between KO and WT mice in the number of mature oligodendrocytes. *N* = 5 WT, 5 KO. Two-way ANOVA: main effect of region, *F*(1,17) = 0.92, *p* > 0.35; main effect of genotype, *F*(1,17) = 2.1, *p* > 0.16; and interaction, *F*(1,17) = 0.18, *p* > 0.6. **(C)** White matter cross-sectional area, evaluated by Myelin Basic Protein (MBP) immunostaining, was elevated specifically in the central striatum. *N* = 6 WT, 6 KO. Two-way ANOVA: main effect of region, *F*(1,25) = 0.53, *p* > 0.45; main effect of genotype, *F*(1,25) = 2.3, *p* = 0.15; interaction: *F*(1, 25) = 4.630; *p* = 0.04. **(D)** Representative micrographs of WT and KO striata immunostained for myelin. All data are presented as mean ± SEM. *p < 0.05 by Sidak’s *post-hoc* test.

Increased RNA and protein of myelin-related genes could either reflect a greater number of oligodendrocytes in the tissue, or a greater amount of myelin production by each oligodendrocytes. To distinguish between these possibilities, we stained mature oligodendrocytes (defined by their expression of CC1) in the dorsal striatum and quantified them by counting all CC1 + cells in four microscope fields in each mouse (two fields, one on each side, from anterior and central dorsal striatum; see Supplementary Figure 6). No significant differences were found between *Hdc*-KO mice and WT controls in overall dorsal striatum or in anterior or posterior subregions; if anything, the trend was toward reduced oligodendrocyte density in KO mice, though this did not reach statistical significance (Figures 3A,B).

To further examine myelin, we immunostained slices for MBP and quantified the total cross-sectional area of white matter, in pixels, in four fields in the dorsal striatum, similarly placed to those used for oligodendrocyte quantification. There was no overall difference in myelin cross-sectional area throughout the striatum, but when we separately examined anterior and central slices a genotype effect emerged, with an increase in cross-sectional area of myelin in the central dorsal striatum of *Hdc*-KO mice (Figures 3C,D).

### Diffusion tensor imaging confirms altered striatal myelin in *histidine decarboxylase*-knockout mice

**FIGURE 4:**
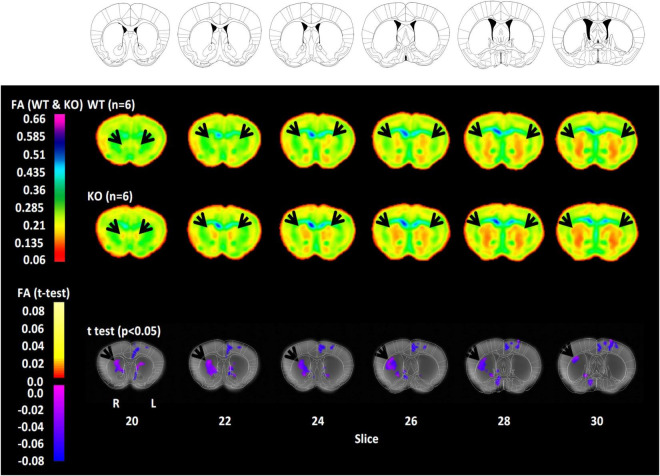
Reduced fractional anisotropy (FA) in the dorsal striatum of *histidine decarboxylase*-knockout (*Hdc*-KO) mice. Top rows: Coronal sections from the reference atlas to which diffusion tensor imaging (DTI) information was registered for analysis. Middle rows: FA in adult male WT and *Hdc*-KO mice (*n* = 6 per genotype). Striatum is indicated by arrowheads. Bottom row: Whole-brain corrected voxel-wise *t*-test comparison revealed reduced FA in the dorsal striatum of *Hdc*-KO mice, more prominently on the right. There were no regions of increased FA in *Hdc*-KO mice. *N* = 6 WT, 6 KO.

Finally, to characterize the impact of these alterations on myelin integrity, we performed whole-brain *ex vivo* DTI. Brains were fixed and imaged *ex vivo* at 9.4 T; voxel-wise FA was computed throughout the brain. While our analysis examined all voxels in the brain, we focused our attention on the dorsal striatum due the molecular and immunohistochemical findings detailed above. Voxel-wise analysis revealed reduced FA in the dorsal striatum of *Hdc*-KO mice (Figure 4). When the statistical threshold was relaxed the effect was seen bilaterally, but it was more prominent and more statistically significant on the right (Supplementary Figure 7). Scattered voxels of between-genotype differences in DTI were seen elsewhere in the brain, but there were no other large contiguous areas of difference.

## Discussion

The *Hdc*-KO mouse recapitulates a rare but high-penetrance mutation associated with TS and exhibits parallels with human disease in behavior, brain activity, neurochemistry, and neurotransmitter receptors (Ercan-Sencicek et al., 2010; Castellan Baldan et al., 2014). These parallels suggest that this model captures important aspects of the pathophysiology of TS and thus allows testing of potential pathophysiological processes using experimental approaches that are not available in humans. We present such an analysis here.

Several genes associated with oligodendroglia and with myelin showed increased expression in the dorsal striatum of *Hdc*-KO mice; this was first seen in RNA-seq analysis and was then confirmed using q-rPCR. Upregulation of myelin-associated genes and abnormal myelination were further confirmed using Western blotting and immunostaining. Oligodendrocyte-associated genes have been implicated in neuropsychiatric disorders, including autism spectrum disorder (ASD) (Phan et al., 2020), but not previously in TS. These changes are region specific: no differences were found in the hypothalamus (RNA-seq) the hippocampus (immunoblot) or the cortex (both). Pathological changes in the striatum have previously been implicated in TS and figure prominently in network models of the pathophysiology of the disorder (Williams et al., 2013).

To examine myelin integrity in the intact brain, we used *ex vivo* MRI imaging and DTI analysis. Due to the high sensitivity provided by high magnetic field DTI (9.4 T) in the murine brain, developmentally and pathologically induced changes in FA can be observed not only in white matter but also in gray matter (Chahboune et al., 2007, 2009a,b; Chua et al., 2009; Carlyle et al., 2012; Duque et al., 2012; Jung et al., 2016; Park et al., 2016; Johnson et al., 2018). There is increasing evidence that myelin is present in gray matter and can be altered there by disease processes (Timmler and Simons, 2019; Long et al., 2021). We found reduced FA in the dorsal striatum of *Hdc*-KO mice. The mouse striatum, unlike the primate caudate and putamen, consists of a combination of gray and white matter, as the heavily myelinated fibers of the internal capsule pass through the gray matter of the striatum. There were no other significant genotype effects on FA (Figure 4). This specificity parallels what we found in our molecular analyses. Abnormal FA has been described before in the basal ganglia system of patients with TS (Neuner et al., 2010; Saporta et al., 2010; Worbe et al., 2015; Martino et al., 2019; Bruce et al., 2021; Yang et al., 2021).

One previous study examined whole-brain morphometry and FA in *Hdc*-KO mice, reporting no abnormalities (Abdurakhmanova et al., 2017). This discrepancy may derive from the modest number of animals examined in both studies (*n* = 5, five in that study; *n* = 6, six here), which may predispose toward false negative results. Our attention was particularly focused on the dorsal striatum because of convergent results from mRNA, protein, and immunohistochemical analyses. Abdurakhmanova et al. (2017) used a volume coil, whereas here we used a surface coil, which provides better signal-to-noise. The discrepancy may also derive from differences in analytic approach, or from subtle differences in the mice examined (genetic background; rearing and housing conditions; age). Male mice were used in both studies. The convergence of DTI findings in the striatum with abnormalities found in parallel molecular and histological analyses in our study increases confidence in the abnormality in myelination in the striatum that we observe here.

Reduced FA may derive from a variety of different abnormalities. It may indicate less myelination, but it can also indicate disordered myelination that constrains water diffusion less than healthy white matter. The use of an animal model allows examination of white matter using a range of methods in parallel, which provides greater clarity. Our finding of reduced FA but increased MBP and MOBP protein and myelin cross-sectional area in the dorsal striatum suggests that, rather than white matter being reduced in *Hdc*-KO mice, myelin integrity is compromised. Hypermyelination can be pathological (Macklin, 2010). For example, in the case of ASD-associated mutations of Pten or MeCP2 (Maire et al., 2014; Sharma et al., 2015), myelin proteins are increased in the context of overall brain dysfunction (Fraser et al., 2008; Vora et al., 2010). That said, the possibility that reduced FA in these mice is attributable to other causes, such as axonal abnormalities, cannot be ruled out.

In both RNA-seq and DTI analyses, white matter abnormalities in *Hdc*-KO mice are limited to the dorsal striatum; no alterations are seen in ventral striatum. DTI analysis, which allows examination of the entire brain, reveals abnormalities restricted the central dorsal striatum (Figure 4). DTI also suggests a greater reduction in FA on the right side; however, trends in the same direction are also seen on the left (see Supplementary Figure 5), and so this apparent lateralization may be a statistical artifact.

This study is cross-sectional, and so the mechanisms and ontogeny of these abnormalities cannot be determined from these data. It is possible that histamine directly regulates oligodendroglia. For example, one study has shown that histamine negatively regulates oligodendrocyte differentiation *via* the H3 receptor, and that the administration of the inverse agonist GSK247246 improves remyelination (Chen et al., 2017). Interestingly, the H3R histamine receptor is elevated in the striatum of *Hdc*-KO mice, and H3R agonists, acting in the dorsal striatum, trigger tic-like movements in the model (Rapanelli et al., 2016). However, the total number of CC1^+^ cells did not differ between *Hdc*-KO and WT animals, suggesting that the increase in myelin proteins is not due to the presence of more mature oligodendrocytes in the striatum, but rather to altered function. Another possibility is that the neuronal activity shapes oligodendrocyte function in the striatum over time. Increasing neuronal activity can stimulate axonal myelination, whereas reducing activity has the opposite effect (Gibson et al., 2014; Mitew et al., 2018). *Hdc*-KO mice have been shown to have elevated neural activity in the striatum (Castellan Baldan et al., 2014). It is possible that chronic elevation in the activity of corticostriatal afferents and/or of striatal neurons leads to increased but disordered myelination over the course of development, resulting in the abnormalities we see here in young adult animals. Future longitudinal analyses may shed light on questions of mechanism and ontogeny.

This study demonstrates the power of combining exploratory molecular analyses–in this case, RNA-seq–with hypothesis-driven multimodal confirmatory follow-up investigations. Further clarification of the precise nature of white matter dysregulation in this animal model may inform future translational work in patients. Complementary investigations in animal models of pathophysiology and in patient samples hold great power to shed new light on pathophysiology.

## Data availability statement

The datasets presented in this study can be found in online repositories. The names of the repository/repositories and accession number(s) can be found in the article/Supplementary material.

## Ethics statement

The animal study was reviewed and approved by the Yale University and Washington University Institutional Animal Care and Use Committees.

## Author contributions

KJ performed molecular and imaging experiments and data analysis, in collaboration with other authors, and wrote the first draft of the manuscript. CY performed RNA-seq analysis. YH and DC performed DTI data collection and analysis. MR supported molecular experiments. FH supervised imaging data collection and analysis and edited the manuscript. JD supervised RNA-seq data collection and analysis and edited the manuscript. LF supported molecular and histological analysis and wrote the manuscript. CP supervised all experiments and data analysis and wrote the manuscript. All authors contributed to the article and approved the submitted version, with the exception of MR.

## Footnotes

1
https://arriveguidelines.org

2
www.ncbi.nlm.nih.gov/geo

3
http://cbl-gorilla.cs.technion.ac.il/

4
bioimagesuite.yale.edu
