# Supplementary material for: White matter abnormalities in the Hdc knockout mouse, a model of tic and OCD pathophysiology
Source: Front Mol Neurosci. 2022 Nov 24;15:1037481. doi: 10.3389/fnmol.2022.1037481 (PMC9731796; doi:10.3389/fnmol.2022.1037481)
Supplement: Supplementary file 1 [file Data_Sheet_1.PDF]

Supplementary information for

White matter abnormalities in the *Hdc* knockout mouse, a model of tic and OCD pathophysiology

Kantiya Jindachomthong, BS[1,§]
Chengran Yang, Ph.D.[6]
Yuegao Huang, Ph.D.[4]
Daniel Coman, Ph.D.[4]
Maximiliano Rapanelli, Ph.D.[1,#]
D.S. Fahmeed Hyder, Ph.D.[4,5]
Joseph Dougherty, Ph.D.[6]
Luciana Frick, Ph.D.[1,†,*]
Christopher Pittenger, MD, Ph.D.[1,2,3,*]

[1]Department of Psychiatry, [2]Yale Child Study Center, [3]Interdepartmental Neuroscience Program, [4]Depatrent of Radiology and Biomedical Imaging, and [5]Department of Biomedical Engineering
Yale University School of Medicine

[6]Department of Genetics, Washington University in St. Louis

[§] Current address: Boston University School of Medicine, Boston, MA

[†] Current address: Department of Neurology and Hunter James Kelly Research Institute, University at Buffalo, Buffalo, NY

[#]Dr. Rapanelli passed away in August, 2020

* Correspondence:
34 Park Street, W315
New Haven, CT 06524
203-974-7675
lfrick@buffalo.edu
christopher.pittenger@yale.edu

Supplementary Figure 1

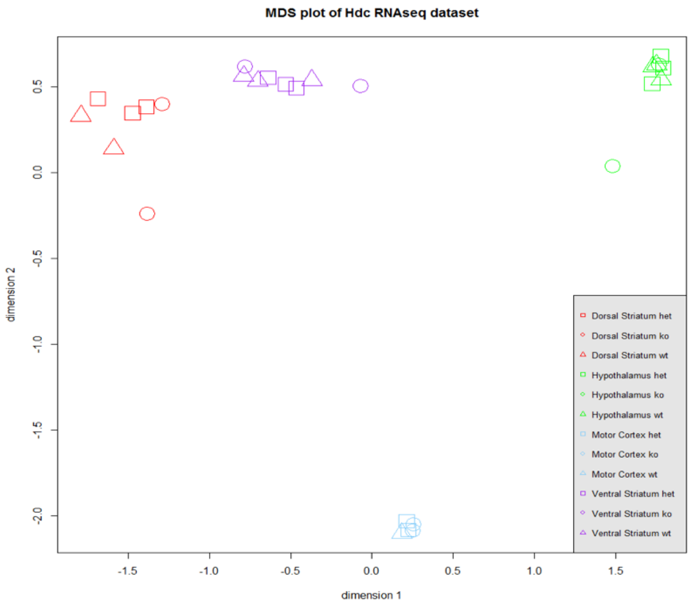

Multidimensional scaling (MDS) analysis of RNA-seq data showed that 28 mRNA samples cluster primarily by tissue, as expected. The colors represent four different tissues (Red is dorsal striatum, green is hypothalamus, blue is motor cortex and purple is ventral striatum). Shapes represent three different genotypes (square is heterozygotes, circle is knockout, triangle is wild-type).

Supplementary Figure 2

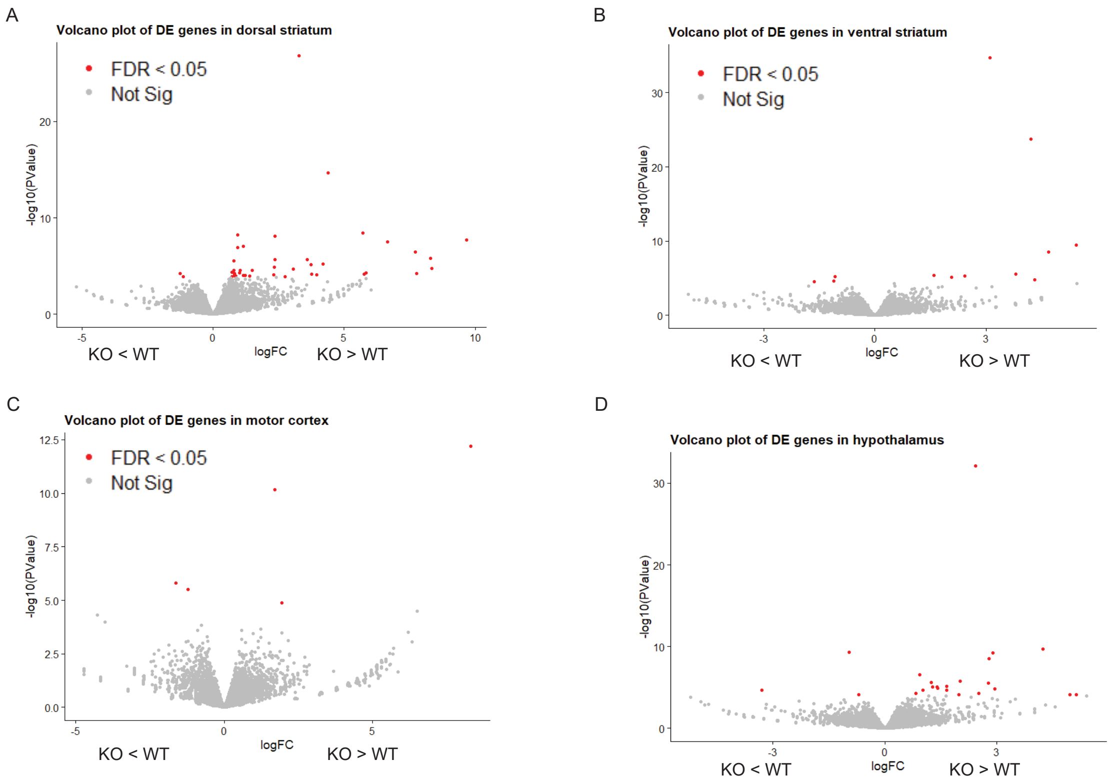

Volcano plots for all differentially expressed genes (KO vs WT). A. DE genes in dorsal striatum. **B.** DE genes in ventral striatum. **C.** DE genes in motor cortex. **D.** DE genes in hypothalamus.

Supplementary Figure 3.

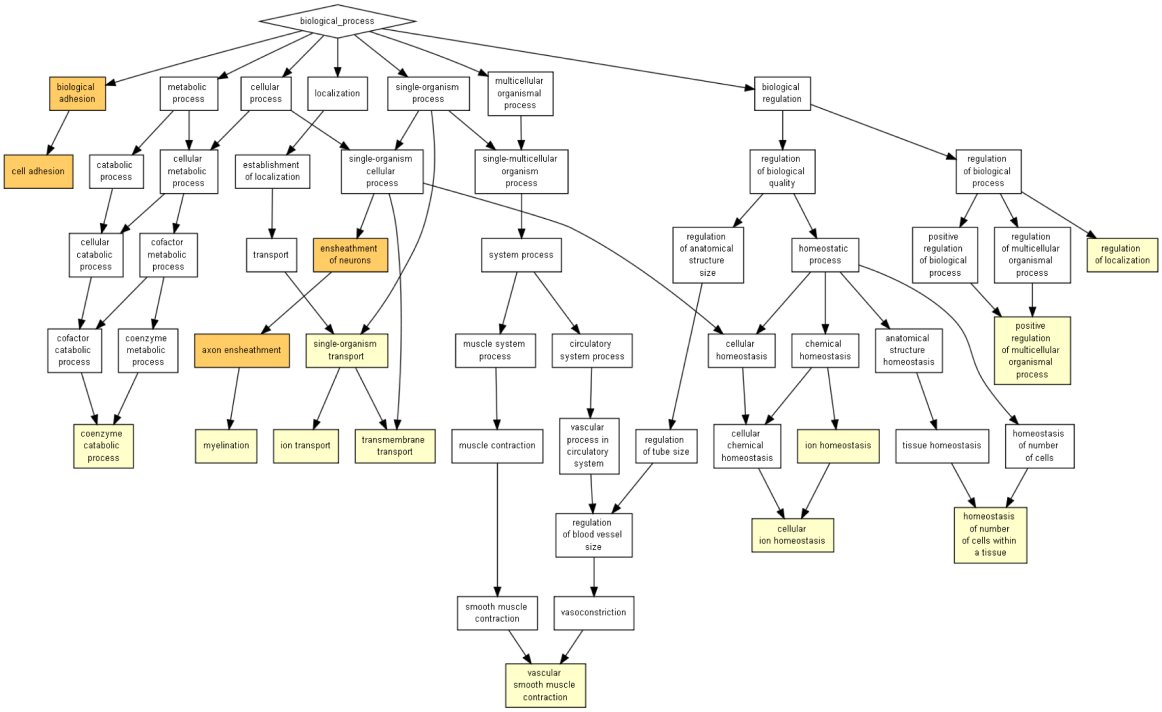

GORILLA analysis of nominally upregulated genes in *Hdc*-KO dorsal striatum, compared to WT (target set n=778, background set n=7376). Output is organized hierarchically by pathway; color corresponds to statistical significance. No statistically significant effects of genotype were found in other regions examined (ventral striatum, hypothalamus, cortex).

Supplementary Figure 4.

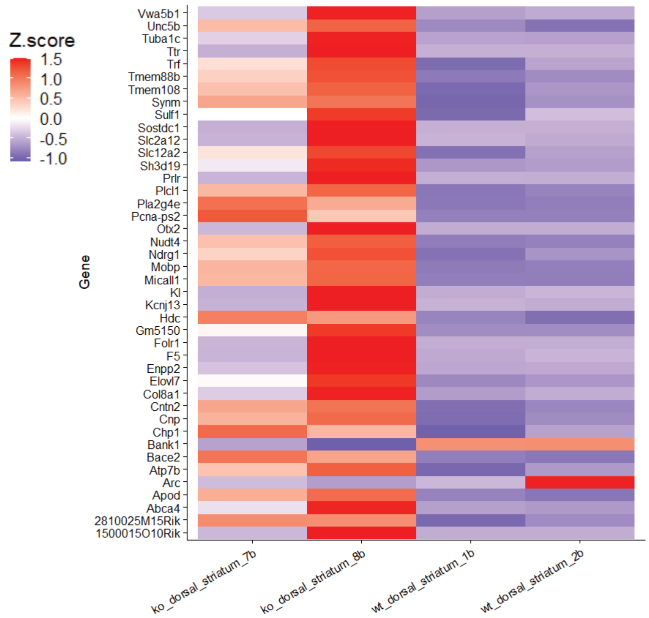

Heatmap of DE genes in dorsal striatum. Z score was calculated for each gene across samples. Oligodendrocyte transcripts were reliably upregulated in DS from KO mice.

Supplementary Figure 5.

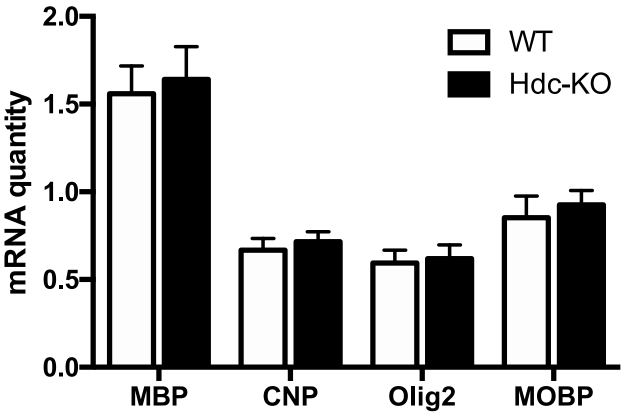

qPCR of myelin-associated genes in whole striatal homogenates showed no differences in expression; this contrasts to the upregulation seen in qPCR from dorsal striatum (see Figure 1D).

Supplementary Figure 6

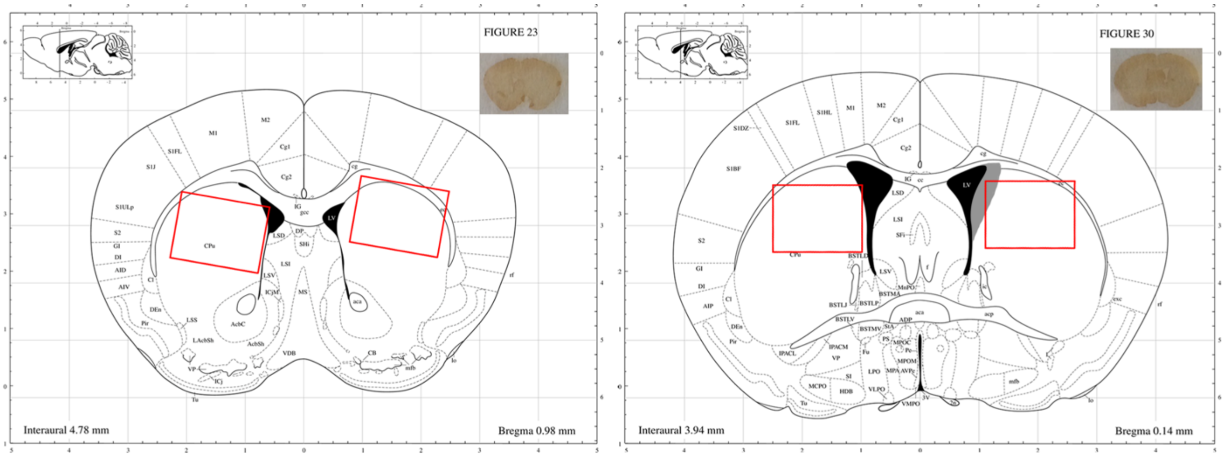

Representative microscope fields used for quantification of oligodendrocytes and of white matter cross-sectional area (Figure 3).

Supplementary Figure 7

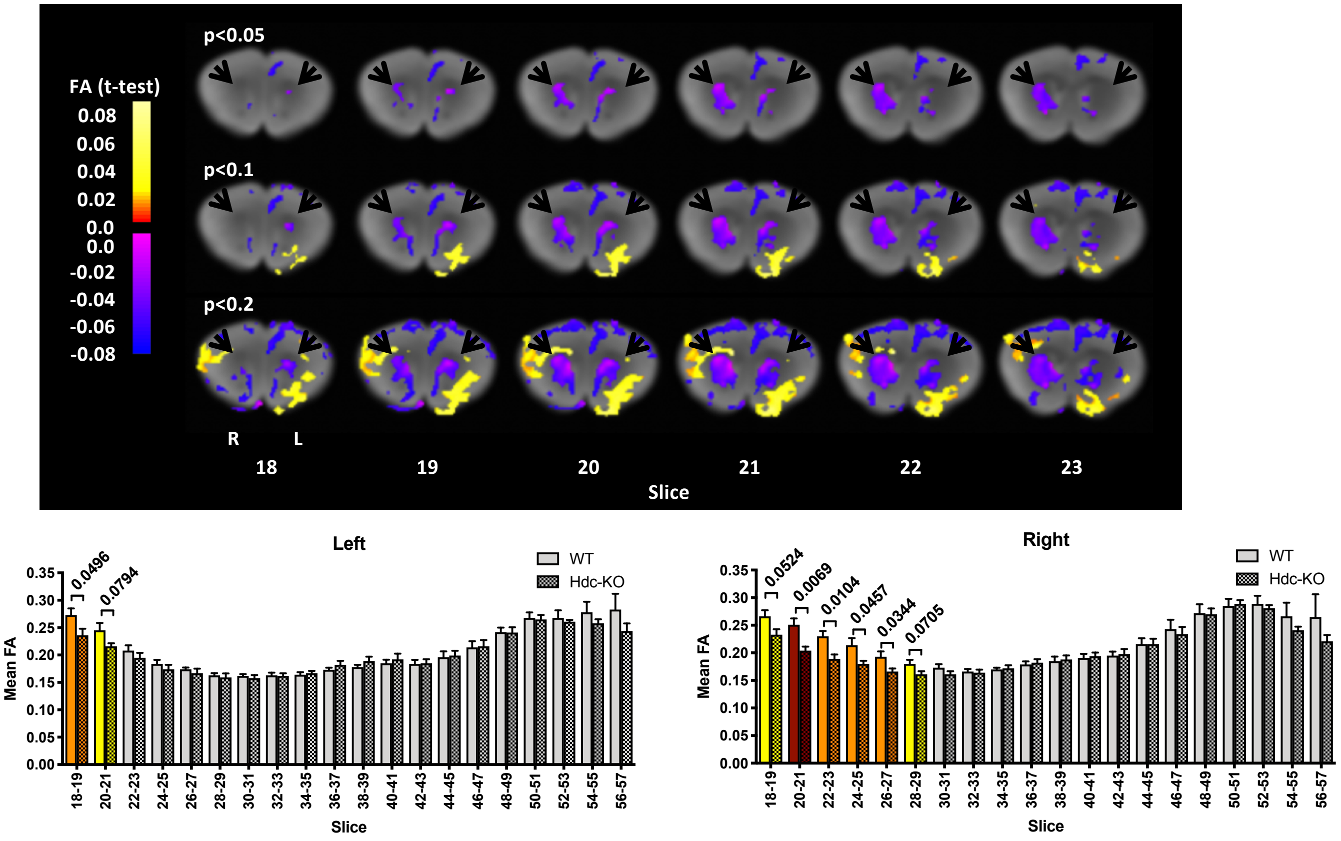

Lateralization of FA reduction in *Hdc*-KO mice. Reduced FA was seen in both hemispheres, but the reduction was both numerically larger and more statistically significant on the right side. When the statistical threshold was relaxed, reduced FA was apparent on the left side, suggesting a symmetrical effect.

Supplementary Table 1

Sample	tissue	genotype	Total reads	Final library size
hdc_het_dorsal_striatum_4b	dorsal_striatum	het	10,565,846	5,025,148
hdc_het_dorsal_striatum_5b	dorsal_striatum	het	10,221,288	4,744,851
hdc_het_dorsal_striatum_6b	dorsal_striatum	het	8,962,236	4,167,862
hdc_het_hypothalamus_4f	hypothalamus	het	10,182,050	4,812,801
hdc_het_hypothalamus_5f	hypothalamus	het	10,133,584	4,761,976
hdc_het_hypothalamus_6f	hypothalamus	het	9,817,236	4,713,427
hdc_het_motor_cortex_5d	motor_cortex	het	9,862,216	4,504,618
hdc_het_motor_cortex_6d	motor_cortex	het	9,110,854	4,137,480
hdc_het_ventral_striatum_4a	ventral_striatum	het	10,613,533	5,061,706
hdc_het_ventral_striatum_5a	ventral_striatum	het	8,886,280	4,019,566
hdc_het_ventral_striatum_6a	ventral_striatum	het	9,649,725	4,172,544
hdc_ko_dorsal_striatum_7b	dorsal_striatum	ko	9,965,035	4,506,374
hdc_ko_dorsal_striatum_8b	dorsal_striatum	ko	8,851,014	3,907,721
hdc_ko_hypothalamus_7f	hypothalamus	ko	9,146,586	3,859,222
hdc_ko_hypothalamus_8f	hypothalamus	ko	9,504,544	4,392,675
hdc_ko_motor_cortex_7d	motor_cortex	ko	9,636,674	4,266,875
hdc_ko_motor_cortex_8d	motor_cortex	ko	8,352,158	3,730,185
hdc_ko_ventral_striatum_7a	ventral_striatum	ko	9,704,408	4,558,826
hdc_ko_ventral_striatum_8a	ventral_striatum	ko	8,449,845	3,711,263
hdc_wt_dorsal_striatum_1b	dorsal_striatum	wt	8,916,839	4,547,116
hdc_wt_dorsal_striatum_2b	dorsal_striatum	wt	11,131,649	5,147,074
hdc_wt_hypothalamus_1f	hypothalamus	wt	10,185,990	4,385,945
hdc_wt_hypothalamus_2f	hypothalamus	wt	9,803,283	4,897,134
hdc_wt_hypothalamus_3f	hypothalamus	wt	10,310,118	4,579,779
hdc_wt_motor_cortex_2d	motor_cortex	wt	9,284,818	4,317,384
hdc_wt_ventral_striatum_1a	ventral_striatum	wt	7,734,808	3,534,939
hdc_wt_ventral_striatum_2a	ventral_striatum	wt	8,962,422	3,935,966
hdc_wt_ventral_striatum_3a	ventral_striatum	wt	8,394,640	3,954,962

Number of mRNA samples used for RNA-seq analysis across three genotypes and four brain regions, after quality control. 'Sample' – unique sample name. 'tissue' – dissected tissue type. 'genotype' – *Hdc* genotype. 'total reads' – original sequencing depth. 'final library size' – library size after alignment, used for subsequent analysis.

Supplementary Table 2 (see attached .xlsx file). **Matrix of counts per million (CPM) for all samples.** 14979 genes as rows, 28 samples as columns.

Supplementary Table 3 (see attached .xlsx file). **Differentially expressed genes (DEGs) in the dorsal striatum. '**Gene_id': Ensembl gene ID. 'gene_name': gene symbol. 'chr': chromosomal location. 'logFC': log2 fold change of KO over WT. 'PValue': unadjusted p-value. 'FDR': adjusted p-value.

Supplementary Table 4

Gene	Forward Primer	Reverse Primer
**MBP**	TCACAGCGATCCAAGTACCTG	CCCCTGTCACCGCTAAAGAA
**CNP**	CAGCTCAAGGAGAAGAACC	TTGTACAGTGCAGCACACC
**OLIG2**	TCCCCAGAACCCGATGATCTT	CGTGGACGAGGACACAGTC
**MOBP**	AACTCCAAGCGTGAGATCGT	CAGAGGCTGTCCATTCACAA

Sequences of the primers used for qPCR analysis of mRNA expression of oligodendrocyte markers (Figure 1C).
